# Sex differences in body composition, metabolism‐related hormones, and energy homeostasis during aging in Wistar rats

**DOI:** 10.14814/phy2.14597

**Published:** 2020-10-19

**Authors:** Susana Quirós Cognuck, Wagner L. Reis, Marcia Silva, Lucas K. Debarba, Andre S. Mecawi, Francisco J.A. de Paula, Celso Rodrigues Franci, Lucila L.K. Elias, Jose Antunes‐Rodrigues

**Affiliations:** ^1^ Physiology Department Ribeirao Preto Medicine School, University of Sao Paulo Ribeirao Preto, Sao Paulo Brazil; ^2^ Department of Physiological Science Center of Biological Sciences Federal University of Santa Catarina Florianópolos Brazil; ^3^ Laboratory of Neuroendocrinology Department of Biophysics Escola Paulista de Medicina Universidade Federal de Sao Paulo Sao Paulo Brazil; ^4^ Medical Clinic Department Ribeirao Preto Medicine School University of Sao Paulo Ribeirao Preto, Sao Paulo Brazil

**Keywords:** aging, body composition, energy metabolism, metabolism‐related hormone, sex differences

## Abstract

Aging affects the body composition and balance of energy metabolism. Here, we collected in a single work several physiological parameters to show how aging and sex differences can influence energy homeostasis. Body mass index (BMI), Lee index, glucose tolerance, glycemia, and lipidogram in fasting were measured in male and female Wistar rats at the ages of 2, 6, 9, 12, and 18 months. We also measured the lipid profile, free fatty acids, glycerol, glycemia, leptin, adiponectin, insulin, corticosterone (CORT), prolactin (PRL), thyroid stimulated hormone, and triiodothyronine (T3) in 3‐ and 18‐month‐old rats of both sexes, fed ad libitum. Animals were classified as obese beginning at 2 months in males and 6 months in females. Aged male rats showed hyperglycemia and glucose intolerance compared to young males and old females. In the ad libitum condition, the 18‐month males presented higher serum levels of triglycerides, total cholesterol, and free fatty acids than females. The 18‐month‐old females had higher PRL and CORT concentration than males, but insulin and T3 were higher in 18‐month‐old males than females. Our work demonstrated that aging processes on energy metabolism in rats is sex specific, with a better lipid profile and glucose tolerance in aged females.

## Graphical abstract

We measured the lipid profile, free fatty acids, glycerol, glycemia, leptin, adiponectin, insulin, corticosterone, prolactin, thyroid stimulated hormone, and triiodothyronine in 3‐ and 18‐month‐old rats of both sexes, fed ad libitum. Our results show that the effects of aging on energy metabolism and endocrine system in rats is sex‐specific, with a better lipid profile and glucose tolerance in aged females.

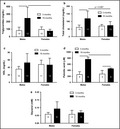

## 1 INTRODUCTION

Males and females have different energy metabolism and susceptibility to pathophysiological conditions, such as obesity and type II diabetes (Lovejoy & Sainsbury, 2009). Sexual differences in body composition, lipid metabolism, and glucose regulation can be modified throughout life or as a function of lifestyle (Ethun, 2016).

Aging changes the body composition, balance between energy availability and demand, signal network that controls homeostasis, and neurodegeneration production. Body composition changes are the most obvious and inevitable effect of aging (Ferrucci et al., 2012; Zamboni et al., 2003).

Changes in the body composition during aging in humans and most mammals include increased adipose tissue, body mass index (BMI), and waist and hip circumference, along with decreased lean body mass and bone mineral density (BMD) (JafariNasabian et al., 2017; Wolden‐Hanson, 2010; Zamboni et al., 2003). However, women have greater body fat and less life‐long lean mass than men, indicating that gender differences remain throughout life (Kirchengast, 2010; Zamboni et al., 2003). On the other hand, older men have higher abdominal fat, serum triglyceride level, and atherogenic index as well as lower high‐density lipoprotein (HDL) cholesterol than older women (Morita et al., 2006; Wu et al., 2001; Zamboni et al., 2003).

Aging and sex modify the secretion of hormones related to energy metabolism, such as glucocorticoids. Stress‐free older female rats have higher plasma corticosterone (CORT) concentration than males, and chronic stress stimuli have no effect on CORT levels in either sex (Bowman et al., 2006). However, in elderly humans, hypothalamus–pituitary–adrenal (HPA) axis activity increases, while daytime release amplitude decreases. Differences were also observed according to sex; where elderly women had higher human corticotrophin‐releasing hormone (CRH)‐stimulated plasma cortisol and adrenocorticotropic hormone (ACTH) concentrations than men (Born et al., 1995; Deuschle et al., 1997).

Other examples are insulin, leptin, and adiponectin secretion, which, when modified, may contribute to the development of obesity‐related diseases, such as diabetes and metabolic syndrome (Basu et al., 2006; Schautz et al., 2012; Schianca et al., 2006). Prolactin (PRL) secretion is also determined by sex, age, and BMI, and high circulating PRL is associated with lower prevalence of diabetes and hypolipidemia (Roelfsema et al., 2012; Wang et al., 2013).

The hypothalamus–pituitary–thyroid axis in healthy elderly women appears to be altered, because plasma triiodothyronine (T3) concentration decreases without altering the secretion of pulsatile thyroid‐stimulating hormone (TSH) (Rossmanith et al., 1992). In addition, the incidence of thyroid diseases, such as hypothyroidism, nodular goiter, and cancer, is highest among postmenopausal and elderly women (Gietka‐Czernel, 2017). Thyroid dysfunction could increase cardiovascular risk, bone fractures, cognitive problems, depression, and mortality (Gietka‐Czernel, 2017).

Life expectancy is crucial in animal research. Rats have a life span of 2–3.75 years, and when treated with reduced‐calorie diets, life expectancy can reach up to 4.6 years (Nistiar et al., 2012; Weindruch & Sohal, 1997). Hence, aging changes in energy metabolism and endocrine system appear to differ by sex and there are few works done in male and female aged rats and each one abord separately lipid and glucose metabolism, and body composition (He et al., 2018; Santos et al., 2014; Trapani & Pallottini, 2010). Here our proposal was in a single work, track aging and sexual differences in energy homeostasis and some hormones involucrate in them, to show a better panorama of energy metabolism according to age and sex. We hypothesized that with aging, female rats are more susceptible to hormonal variations that produce unbalance the energy metabolism, which are evidenced by body changes. Thus, our goal was to assess body composition, energy metabolism, and hormone secretion at different ages of male and female rats.

## 2 MATERIALS AND METHODS

### 2.1 Animals

We used male and female Wistar rats obtained from the animal facility located at the University of Sao Paulo on the Ribeirao Preto Campus, Brazil. Rats were subjected to experiments at 2, 6, 9, 12, and 18 months old, and another group of rats at age 3 and 18 months old. At 18 months, the female rats were in reproductive senescence. Rats were maintained under controlled temperature of 23 ± 2°C and exposed to a 12:12 hr light–dark cycle (light period: 06–18 hr). The animals were fed with a standard diet (QuimtiaNuvilab®—3.86 kcal/g containing 4% lipids, 22% proteins, and 60% carbohydrates) and water ad libitum. Animals were housed in groups of 5 until they reached the body weight of 500 g, after that, they were housed two rats/cage (cage size: 33.4 × 12.9 × 8.6 inches). All experiments were performed in the morning (from 08 hr to 11 hr), except those to determine the body composition, that was performed between 16 and 17 hr. This study was conducted according to the “Guide for the Care and Use of Laboratory Animals” (NIH; Publication No. 85‐23, revised 1996), and the experimental protocols were approved by the Ethical Committee for Animal Use of the School of Medicine of Ribeirao Preto, University of Sao Paulo (protocol # 014/2014‐1).

In order not to submit the animals to new stress in a short period, the data of the animals that were not collected or were lost during an experiment were not repeated, therefore the number of animals per age is not constant in all experiments and may vary in each age.

### 2.2 Survival rate and food intake

One group of 25 male and 38 female rats were used only to determine the survival rate, we registered the number of dead animals per week starting at the age of 7 weeks old. To determine the food intake, another group of 14 male and 12 female rats were used and kept in metabolic cages for adaptation of 3 days followed by 24 hr food intake measurements.

### 2.3 BMI, Lee index, BMC, BMD, lean and fat mass, and fat percentage

Animals at age of 2, 6, 9, 12, and 18 months were anesthetized with ketamine (50 mg/kg) and xylazine (10 mg/kg) by intraperitoneal injection. Naso‐anal length was measured for later calculation of body mass index (BMI calculated by dividing the weight [g] by the length^2^ [cm^2^]) and Lee index [calculated as cube root of body weight [g]/length [cm]) (Novelli et al., 2007). Subsequently, BMC and BMD, lean and fat mass, and fat percentage were determined using DEXA (Hologic Discovery TM QDR Series, Software: Hologic Discovery Wi Small animals) at the Ribeirao Preto Medical School University Hospital.

### 2.4 Oral glucose tolerance test

To perform the oral glucose tolerance test, animals at age of 2, 6, 9, 12, and 18 months were kept without food for 14 hr with free access to tap water. Fasting glucose level was determined by a drop of blood collected through an incision made at the tip of the animal's tail. After that, animals received by oral gavage, 2 g/kg of glucose diluted in 60 ml of distilled water. Blood glucose was determined at 15, 30, 60, 90, and 120 min. A glucometer and reactive strips for glucometer (Accu‐Check Performa) were used to determine the glycemic values, which were used to calculate the areas under the curve.

### 2.5 Lipidogram, free fatty acids, glycerol, and glycemia

For the lipidogram, animals at age of 2, 6, 9, 12, and 18 months were kept in fasting for 14 hr, with tap water ad libitum. After this time, blood was collected by an incision at the tip of the rat's tail. Another group of 3‐ and 18‐month‐old male and female rats, kept with water and fed ad libitum, were euthanized by decapitation and blood sample was obtained to determine blood glucose and serum hormone concentrations. Due to the changes along the estrous cycle, only females in diestrus were used.

The determinations of triglycerides, total cholesterol, and HDL cholesterol were performed according to commercial kit manufacturer (Triglycerides Liquiform (REF.: 87), Cholesterol Liquiform (REF.: 76), Cholesterol HDL (REF.: 13), Labtest Diagnóstica, SA, Brazil). All quantifications were performed in triplicate. Free fatty acid and glycerol quantification was made by commercial colorimetric kit (EnzyChromTM Free Fatty Acid Assay [EFFA‐100], BioAssay Systems, and Glycerol Assay Kit [MAK117]), Sigma‐Aldrich‐®, respectively), following the manufacturer's specifications.

### 2.6 Hormonal extractions and immunoassays

Male and female rats aged 3 and 18 months with food and water ad libitum were decapitated and trunk blood was collected in chilled tubes that contained or not heparin (10 μl/ml of blood) to obtain plasma and serum, respectively.

Commercial ELISA kits were used to measure leptin and adiponectin (EMD Millipore Corporation, Billerica, MA, USA), insulin (Alpco, Salem, NH, USA), TSH (Crystal Chem, Grove Village, IL, USA) and T3 concentration (MyBioSource, San Diego, CA, USA).

CORT was extracted from 25 μl of plasma with 1 ml of ethanol. The hormone measurements were performed using specific radioimmunoassay (RIA) techniques described in the literature (Glucocorticoids et al., 1978). All measurements were performed in duplicate in the same assay. The sensitivity of the assay and the coefficient of variation intra‐assay were, respectively, 7.8 ng/dl and 4.6% for CORT.

Plasma prolactin (PRL) concentration was determined by RIA, using antibody provided by the National Hormone and Peptide Program (Harbor‐UCLA Medical Center, CA, USA). The lowest limit of detection was 0.10 ng/ml, intra‐assay coefficient of variation was 2.5%.

### 2.7 Statistical analysis

Data are shown as means (standard deviation [*SD*]). Survival analyzes were performed using the Kaplan–Meier method. Shapiro–Wilkʼs W test was used to determinate the normality of data. Statistical significance of the difference between the means of the studied groups was assessed by two‐way ANOVA followed by Newman–Keuls posttest when the variances were equal, or by Games–Howell analysis when the variances were different. For experiments that were performed over the life of the animals or those that were done over a period, repeated measures ANOVA was used followed by Newman–Keuls posttest; however, when the data did not adjust to normality, Friedman ANOVA and Mann–Whitney *U* test were used. Paired t‐student analysis and unpaired t‐student independent samples were used when appropriate. Significance level of *p* < .05 (two‐tailed) was adopted. Number (*n*) of animals used in each experiment is indicated in the figure legend.

## 3 RESULTS

### 3.1 Survival rate and food intake

**FIGURE 1:**
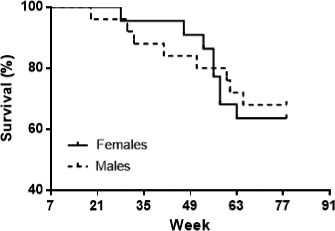
Survival percentage of male and female Wistar rats from 7‐ to 78 weeks of age. Initial and final number of male rats was 25 and 17; and for female rats was 22 and 14, respectively. Kaplan–Meier method

Figure 1 charts survival of the male and female rats at different ages. The 90% survival rate was reached at 31 weeks of age in males and at 47 weeks of age in females. At 78 weeks of age (18‐month‐old), the survival rate for males was 68% and for females 63.6%. No significant differences in mortality were observed between males and females. The death rate for females was 36.4% and males 32% during the evaluated time.

As shown in Table 1, food and energy intake decreased with aging, where the food and energy intake were higher in 2‐month‐old rats than 12‐month‐old animals (Age: food intake: Males *t*_13_ = 4.45; *p* < .001; Females: *t*_6_ = 3.31; *p* < .05 and energy intake: Males *t*_13_ = 4.45; *p* < .001 and females: *t*_6_ = 3.31; *p* < .05). In addition, differences between the sexes were observed at 12 months, where females have both food and energy intake higher than male rats (Sex: food intake: *t*_12_ = 2.94; *p* < .05; energy intake: *t*_12_ = 2.94; *p* < .05). Unfortunately, we could not have food and energy in aged males rats, however, Table 1 shows a continuous diminishing food and energy intake in males rats that we can speculate to be significantly different from females, as it was observed before from Wolden‐Hanson (2006). The body weight for the calculation of food and energy intake is also show in Table 1, where 2‐month‐old rats have lower weight than 12‐month‐old animals (Age: Males *t*_13_ = −11.64; *p* < .00001; Females: *t*_6_ = −6.28; *p* < .001) and male rats are heavier than female rats (Sex: 2‐month‐old: *t*_18_ = −9.33; *p* < .00001 and 12‐month‐old: *t*_12_ = −6.44; *p* < .0001).

**TABLE 1:** Food and energy intake in 24 hr of male (M) and female (F) rats at ages 2, 6, 9, and 12 months, and only in 18‐month‐old females

Parameters	Sex	Age (months)				
		2	6	9	12	18
Food intake (g/100 g bw)	M	6.7 (1.7)	3.8 (0.6)	3.7 (0.9)	3 (1.3)^a^	–
	F	8 (1.3)	5.6 (1.2)	5.8 (2.4)	5.1 (1.3)^a^	5.9 (1.5)
Energy intake (KJ/100 g bw)	M	108.6 (27.5)	61.1 (9.3)	59.8 (14)	49.1 (21.6)^a^	–
	F	129 (21)	89.7 (19.5)	94.2 (38.1)	83 (21.3)^a^	95.6 (23.7)
Body weight (g)	M	448.9 (40.8)	674.1 (81.6)	722.1 (66)	767.2 (65.7)^a^	–
	F	317.2 (21)^c^	421 (41.3)	448.5 (51.7)	499.5(84.1)^c^	548.8 (32.4)
Number of animals	M	9	8	9	6	–
	F	11	9	8	8	4

Note

Values are expressed as means (*SD*).

^a^ Different from 2‐month‐old males *p* < .05.

^b^ Different from 2‐month‐old females *p* < .05.

^c^ Different from same‐age males *p* < .05. Paired *t*‐Student analysis, and unpaired *t*‐Student independent samples (2 and 12 months).

### 3.2 Obesity index over the lifetime of rats

Obesity indexes are shown in Table 2. The BMI was higher in males than females at the age of 6, 9, 12, and 18 months (Sex: *F*_1;12_ = 37.97; *p* < .0001). The 2‐month‐old rats had the lower BMI than the other ages analyzed (Age: *F*_4;48_ = 5.88; *p* < .001). Lee index was higher in males than females at ages 2, 6, 9, and 12 months (Sex × Age Interaction: *F*_4;48_ = 8.14; *p* < .0001).

**TABLE 2:** Body weight, body mass (BMI) and Lee index, bone mineral quantity (BMC), bone mineral density (BMD), lean mass, fat mass, and fat percentage of male and female rats at the ages of 2, 6, 9, 12, and 18 months

Parameters	Sex	Age (months)				
		2	6	9	12	18
BMI (g/cm^2^)	M	0.80 (0.06)	1.07 (0.91)^a^	1.04 (0.08)^a^	1.01 (0.06)^a^	0.95 (0.08)^a^
	F	0.67 (0.03)	0.77 (0.05)^f^	0.79 (0.07)^f^	0.80 (0.08)^f^	0.75 (0.28)^f^
Lee index (g^1/3^/cm)	M	0.34 (0.02)	0.35 (0.01)^a^	0.34 (0.01)^b^	0.33 (0.01)^b^	0.32 (0.01)^a^, ^b^, ^h^
	F	0.31 (0.01)^f^	0.32 (0.01)^f^	0.32 (0.01)^f^	0.32 (0.01)	0.32 (0.01)
Body weight (g)	M	459.8 (67.8)	738.8 (54.8)^a^	771.8 (33.7)^a^	805.8 (49.6)^a^	770.8 (105.2)^a^
	F	347.6 (21.4)^f^	446.4 (37.8)^f^	467.3 (53.8)^f^	516.1 (62.0)^f^	548.8 (54.6)^f^
BMC (g)	M	–	12.4 (7.0)	17.3 (0.6)^b^	18.7 (2.7)^b^	18.4 (3.2)^b^
	F	–	11.2 (1.1)^f^	11.5 (1.2)^f^	16.6 (1.7)^f^	14.7 (1.4)^f^
BMD (g/cm^2^)	M	–	0.15 (0.08)	0.20 (0.00)^b^	0.20 (0.01)^b^	0.20 (0.02)^b^
	F	–	0.18 (0.01)	0.18 (0.01)	0.22 (0.01)^b^	0.20 (0.01)^b^
Lean mass (g)	M	–	515.2 (62.1), *n* = 4	528.2 (32.8), *n* = 4	562.2 (59.3)^b^, *n* = 4	495.4 (36.5)^i^, *n* = 4
	F	–	302.8 (39.0)^f^	310.9 (34.0)^f^	356.0 (63.0)^f^	344.9 (30.5)^f^
Fat mass (g)	M	–	139.6 (35.5), *n* = 4	528.2 (32.8)^b^, *n* = 4	178.4 (24.4)^h^, *n* = 4	179.5 (97.7)^h^, *n* = 4
	F	–	92.8 (22.8)	310.9 (34.0)^h^	115.2 (47.9)^e^	146.7 (30.6)^e^
Fat (%)	M	–	21.4 (5.7), *n* = 4	24.2 (1.9), *n* = 4	24.1 (3.2), *n* = 4	25.5 (10.0), *n* = 4
	F	–	23.5 (5.2)	22.9 (3.3)	24.1 (7.5)	29.7 (4.4)
Number of animals	M	5	5	5	5	5
	F	9	9	9	9	9

Note

Values are expressed as means (*SD*).

^a^ Different from 2‐month‐old males *p* < .05.

^b^ Different from 6‐month‐old males *p* < .05.

^c^ Different from 2‐month‐old females *p* < .05.

^d^ Different from 6‐month‐old females *p* < .05.

^e^ Different from 9‐month‐old females *p* < .05.

^f^ Different from same‐age males *p* < .05.

^g^ Different from 12‐month‐old females *p* < .05.

^h^ Different from 9‐month‐old males *p* < .05.

^i^ Different from 12‐month‐old males *p* < .05. Repeated measures ANOVA followed by Newman–Keuls.

### 3.3 Changes in body composition with age

Table 2 presents the parameters used to determine body composition at different ages according to sex. Body weight was different in all ages according to sex, with males presenting greater body mass than females (Sex: *F*_1;48_ = 90.02; *p* < .00001). The lowest body weight was measured for both males and females at the age of 2 months and highest at the age of 18 months (Age: *F*_4;48_ = 129.24; *p* < .00001).

Males had higher bone mineral content (BMC) than females at the age of 6, 9, and 18 months (Sex: *F*_1;33_ = 25.98; *p* < .001). In addition, 18‐month‐old males showed higher BMC than when they were younger, whereas females exhibited higher BMC at the age of 12 months, and at 18 months their BMC decreased (Age: *F*_3;33_ = 3.96; *p* < .05).

Both males and females at 6 months had lower BMD than when they were 12 and 18 months old (Age: *F*_3;33_ = 36.58; *p* < .00001). The highest BMD was observed in females at age 12 months and males at 18 months. Differences by sex were not observed for BMD.

Males had heavier muscle tissue than females at the ages of 6, 9, 12, and 18 months (Sex: *F*_1;33_ = 82.17; *p* < .00001). At the age of 12 months, both males and females had greater lean mass than when they were 6 months old, and at 18 months, males showed a decline in muscle mass compared to 12 months (Age: *F*_3;33_ = 5,44; *p* < .01).

At 9 months, males presented more fat mass than females (Sex: *F*_1;33_ = 31,52; *p* < .001). At age 18 months, both males and females had greater fat mass than at 9 months of age (Age: *F*_1;33_ = 158.31; *p* < .00001). For the fat percentage, no difference was observed according to sex or age.

### 3.4 Changes in carbohydrate metabolism with age

**FIGURE 2:**
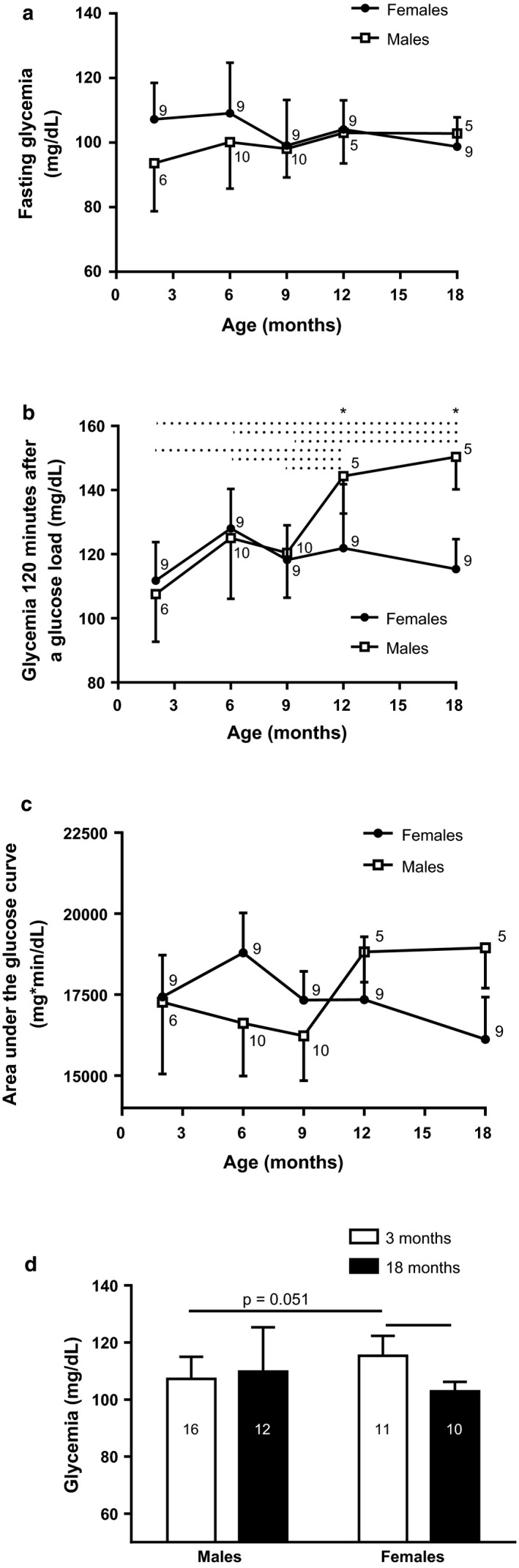
Fasting glycemia (a), glycemia 120 min after a glucose load (b), and area under oral glucose curve (c) of males and females at the ages of 2, 6, 9, 12, and 18 months. Glycemia in 3‐ and 18‐month‐old fed male and female rats (d). Data are shown as means (*SD*), * *p* < .05, comparing by sex, (...........) *p* < .05 between the indicated ages for males. Repeated measures ANOVA followed by Newman–Keuls posttest and two‐way ANOVA followed by Games–Howell posttest. Number (n) for males and females in each month is next to respective shape or in the column

Rats at different ages in fasting condition exhibited no changes in glycemia or differences by sex (Figure 2a). Glycemia 120 min after a glucose load was higher in 12‐ and 18‐month‐old males than females at the same age (Sex × Age Interaction: *F*_4;32_ = 8.99; *p* < .0001). In addition, 12‐ and 18‐month‐old males had higher glycemia than younger ages, and, the highest glycemia was observed in 18‐month‐old males (Age: *F*_4;32_ = 7.52; *p* < .001; Figure 2b). No changes were observed on area under glucose curve of rats at different ages or by sex (Figure 2c). For glycemia in fed rats, 3‐month‐old females displayed higher glycemia than the 18‐month‐old females and 3‐month‐old males (Interaction Sex × Age: *F*_1;45_ = 7.07; *p* < .05 Figure 2d).

### 3.5 Changes in lipid metabolism with age

**FIGURE 3:**
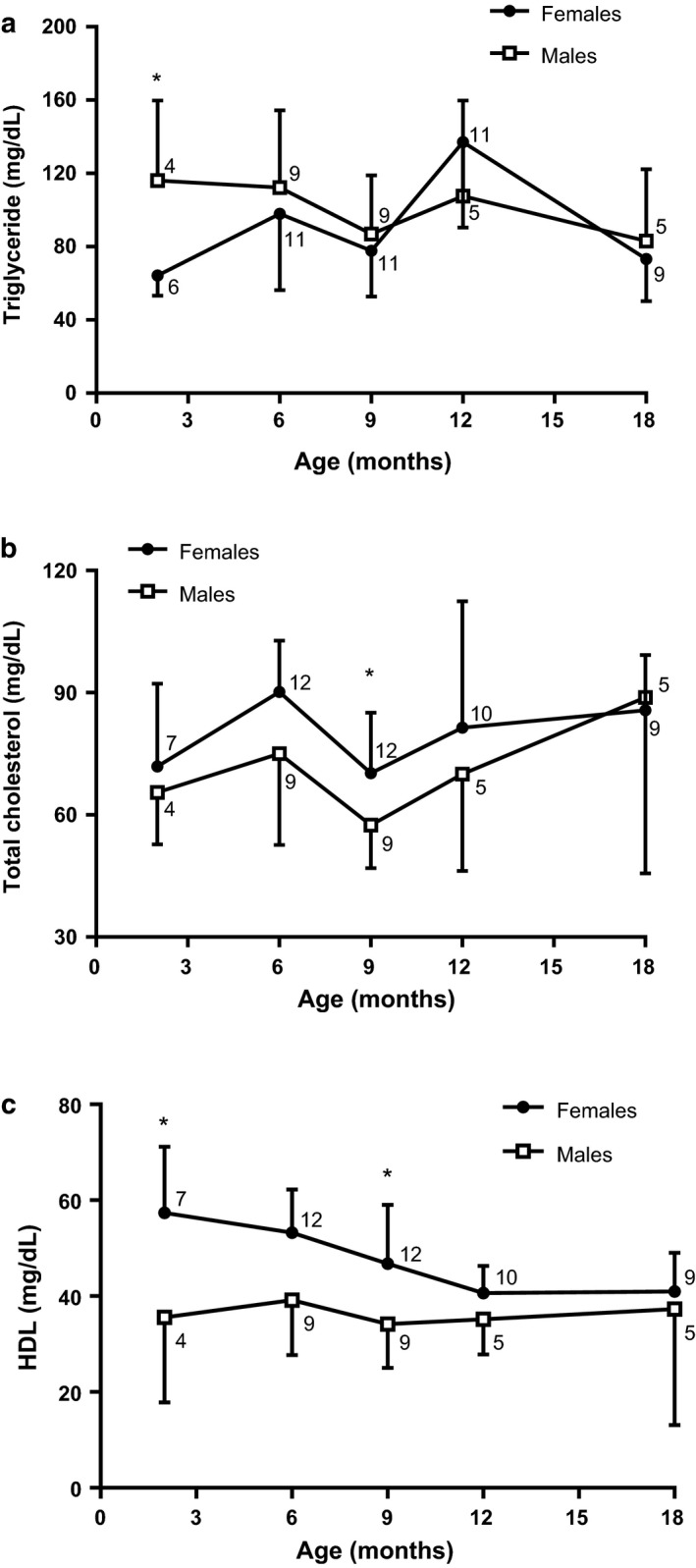
Fasting analysis for triglycerides (a), total cholesterol (b) and HDL cholesterol (c) of 2, 6, 9, 12, and 18‐month males and females. Data are presented as means (*SD*), *p < .05, comparing by sex. Mann–Whitney *U* test, paired t‐student analysis, unpaired *t*‐Student independent samples (2 and 9; 9 and 18 months). *n* for males and females in each month is next to respective shape

Fasting lipid metabolic analysis found that only at 2 months of age, fasting males showed a higher triglycerides concentration than females (Sex: *Z* = −2.45; *p* < .05). Over time, no changes in triglyceride levels were observed in both fasting male and female rats (Figure 3a). For total cholesterol in fasting rats, 9‐month‐old females displayed higher cholesterol than the males (Sex: *t*_19_ = 2.18; *p* < .05; Figure 3b). HDL cholesterol concentration was observed higher in 2‐ and 9‐month‐old females than respective males (Sex: *t*_9_ = 2.28; *p* < .05 and *t*_19_ = 2.58; *p* < .05, respectively). No differences were observed by age in the HDL cholesterol (Figure 3c).

**FIGURE 4:**
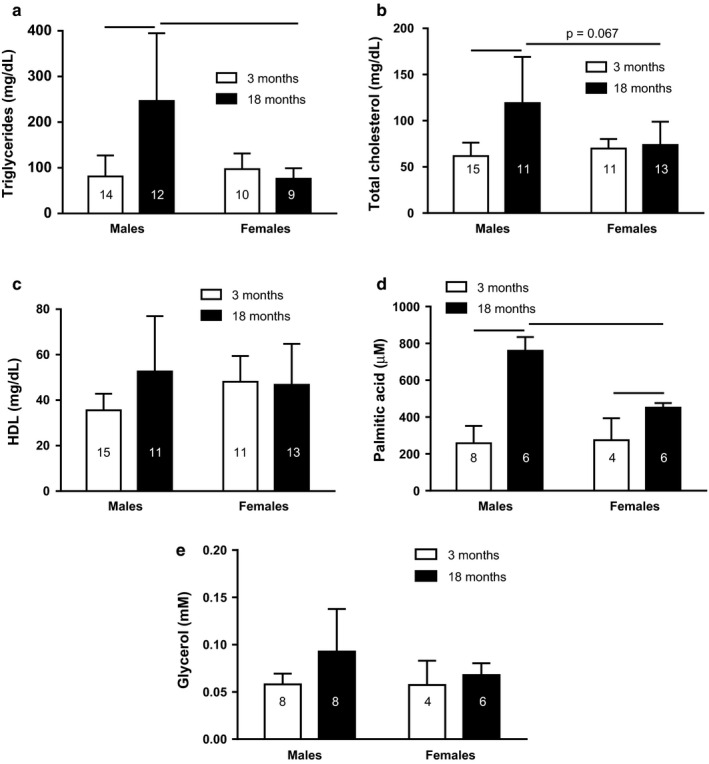
Fed analysis for triglycerides (a), total cholesterol (b), HDL cholesterol (c), free fatty acids (d), and glycerol (e) of 3‐ and 18‐month fed male and female rats. Data are presented as means (*SD*), (_____) *p* < .05 among the indicated groups. Two‐way ANOVA followed by Games–Howell or Newman–Keuls posttest. *n* for each group is in the column

Lipid metabolic analysis in 18‐month‐old male rats in ad libitum conditions found higher triglycerides, total cholesterol, and palmitic acid serum concentration than 3‐month‐old males and 18‐month‐old females (Triglycerides: Sex × Age Interaction: *F*_1;41_ = 13.68; *p* < .01; Figure 4a; Total cholesterol: Sex × Age Interaction: *F*_1;45_ = 10.60; *p* < .01; Figure 4b; and Palmitic acid: Sex × Age Interaction: *F*_1;20_ = 21.92; *p* < .001; Figure 4d).

No differences were observed by sex and age in serum HDL cholesterol and glycerol concentration (Figure 4c,e).

### 3.6 Hormonal changes with age in fed animals

**FIGURE 5:**
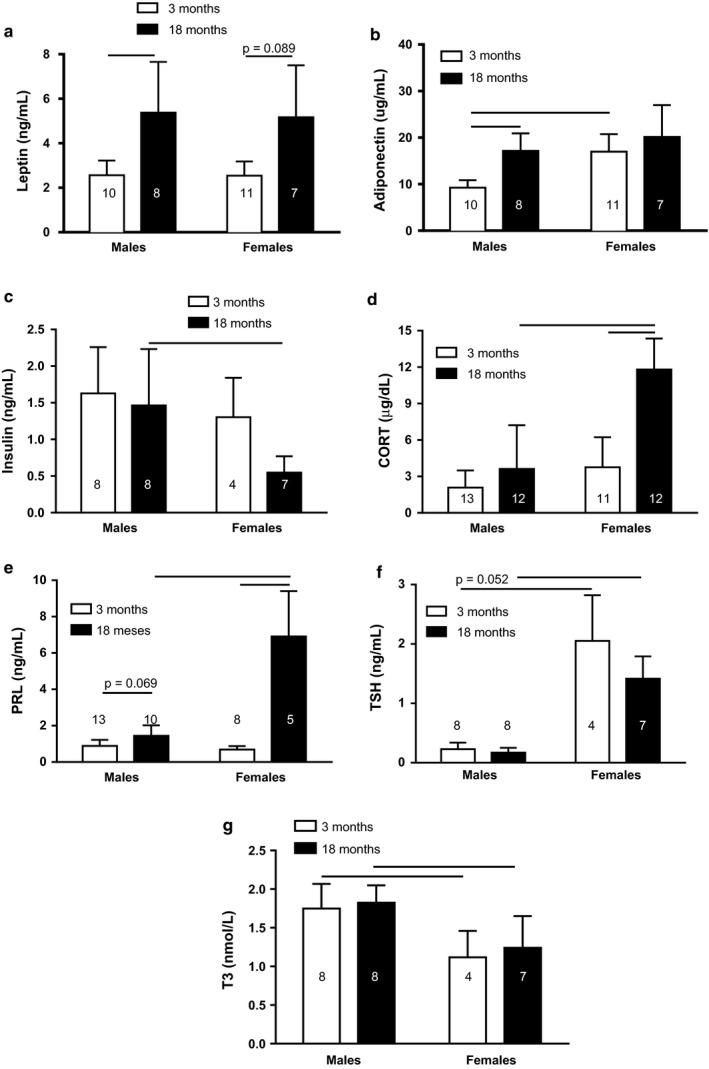
Leptin (a), adiponectin (b), insulin (c), CORT (d), PRL (e), TSH (f), and T3 (g) plasma concentration of 3‐ and 18‐month male and female rats. Data are presented as means (*SD*), (_____) *p* < .05 among the indicated groups. Two‐way ANOVA followed by Games–Howell or Newman–Keuls posttest. n for each group is inside or at the top of the column

Plasma leptin concentration in both male and female rats was higher at 18 months than at 3 months (Age: *F*_1;32_ = 26.78; *p* < .0001; Figure 5a). Plasma adiponectin level was higher in 3‐month‐old females than in 3‐month‐old males (Sex: *F*_1;32_ = 14.62; *p* < .001). In addition, the 18‐month‐old males had higher plasma adiponectin than 3‐month‐old males (Age: *F*_1;32_ = 15.62; *p* < .001; Figure 5b).

Plasma insulin concentration was higher in 18‐month‐old males than in 18‐month‐old females (Sex: *F*_1;23_ = 6.80; *p* < .05; Figure 5c). CORT was higher in 18‐month‐old females than in 3‐month‐old females (Age: *F*_1;44_ = 40.45; *p* < .0001). In addition, the 18‐month‐old females had higher plasma CORT levels than 18‐month‐old males (Sex: *F*_1;44_ = 42.83; *p* < .0001; Figure 5d). Figure 5e shows that plasma PRL concentration was much higher in 18‐month‐old females than in 3‐month‐old females (Age: *F*_1;32_ = 99.31; *p* < .0001) and males at 18 months old (Sex: *F*_1;32_ = 59.88; *p* < .0001).

On the other hand, plasma TSH level was higher in both 3‐ and 18‐month‐old females than in respective males (Sex: *F*_1;23_ = 122.16; *p* < .0001; Figure 5f). However, plasma T3 was lower in both 3‐ and 18‐month‐old females than the respective males (Sex: *F*_1;23_ = 28.68; *p* < .0001; Figure 5g).

## 4 DISCUSSION

Males and females have different energy metabolism, and this can be modified throughout the life (Carrascosa et al., 2009; Ethun, 2016). The current study assessed survival expectancy, body composition, glucose and lipid metabolism, and hormone secretion at different ages of male and female rats. Animals were classified as obese beginning at 2 months in males and 6 months in females. Aged male rats showed hyperglycemia and glucose intolerance compared to young males and old females. In the fed ad libitum condition, the 18‐month‐old males presented higher serum levels of triglycerides, total cholesterol, and free fatty acids than females. The 18‐month‐old females had higher PRL, COR, and TSH concentration than males, but insulin and T3 were higher in 18‐month‐old males than female rats.

Survival expectancy in male Wistar rats varies from 64 to 160 weeks, and no data are available for female Wistar rat. However, for Wiesner and Sheard rats, the 90% survival was achieved at week 71 (Nistiar et al., 2012; Wiesner & Sheard, 1934). In our study, 90% survival rate was reached at week 31 in males and at week 47 in females (Figure 1). Differences found in the survival percentage between our results and those described in the literature may be due to different conditions that affect the life expectancy of animals, including the nutritional composition of food, the rat colony, and the availability for the animals to perform physical activity/inactivity, which could develop obesity.

The rats in our study were generally obese. The BMI, whose normal value for a rat ranges from 0.45 to 0.68 g/cm^2^, was above 0.8 g/cm^2^, indicating that our male animals were obese beginning 2‐months old (Novelli et al., 2007). On the other hand, females presented obesity beginning at 6 months. The Lee index, which states that values above 300 indicate obesity, confirms the BMI results for obesity in our animals (Bernardis, 1970; Novelli et al., 2007).

The largest increase in body weight of male rats, which was observed between the ages of 2 and 6 months, may be associated with somatic growth, because in animals older than 48 days, weight gain is associated with the addition of new cells and enlargement of fat and muscle tissue (Enesco & Leblond, 1962). Thus, the difference observed between males and females in body weight is associated with the effect of sex hormones, where testosterone favors the development of muscle tissue (Bhasin et al., 2003; Singh et al., 2003).

As the animal ages, the cell proliferation in the body slowly decreases and fat storage progressively increases (Enesco & Leblond, 1962). The decrease in lean mass and the increase in fat at the age of 18 months observed in our results may be related to the decrease in testosterone that occurs in older animals, and with it the stimulation in muscle tissue and inhibition in adipose tissue (Rosario et al., 2009; Singh et al., 2003; Smith et al., 1992).

Sex differences observed in food and energy intake may be due to the effects of sex hormones, because estrogen decreases meal size while testosterone increases it (Asarian & Geary, 2006, 2013; Blaustein & Wade, 1976; Chai et al., 1999; Roy & Wade, 1977). Therefore, the higher food intake in 12‐month‐old female rats than males may be related to decreased estrogen and testosterone during aging (Lu et al., 1998; Rosario et al., 2009; Smith et al., 1992).

Sex differences found in BMC is mediated by testosterone, which promotes male growth, acting on both bone size and mass, while estrogens limit the female growth of the appendicular skeleton (Zhang et al., 1999). Also, estradiol stimulates osteoblast proliferation and differentiation and inhibits osteoclast differentiation, so the decrease of BMC and BMD in 18‐month‐old female rats may be due to decreased estrogen and high CORT levels, which reduce maturation, lifespan, and function of osteoblast (Cannarella et al., 2019). On the other hand, the fact that at 18‐month‐old male rats kept BMD could be because estrogen is a major regulator of bone in male, and estradiol levels do not change in the aged male (Greenblatt et al., 1976; Khosla & Monroe, 2001; Van Pottelbergh et al., 2003). Although the testosterone level decrease with aging, the aromatase enzyme, that converts androgens into estrogens, activity increase (Van Pottelbergh et al., 2003; Vermeulen et al., 2002).

The increase in plasma blood glucose levels observed in 12‐ and 18‐month‐old male rats (Figure 2b) may be due to the interaction of four factors: decreased insulin production, decreased insulin sensitivity, increased glucagon production, and alteration in the expression of glucose transporter (GLUT)4 in skeletal muscle by estradiol. Elderly rats have reduced proliferation and increased frequency of pancreatic β‐cell apoptosis, which is associated with age‐related pancreatic β‐cell dysfunction (Gu et al., 2012). In addition, 17‐month‐old Wistar rats have impaired insulin sensitivity and 17‐ and 18‐month‐old males submitted to an oral glucose load 2 hr later exhibited increased plasma glucagon values (Muñoz et al., 2018; Yoshino et al., 1979). Also, in the skeletal muscle of male mice predominate the estrogen receptor beta, that reduces the expression of the GLUT4 (Barros et al., 2006, 2009).

Different studies in female rats submitted to ovariectomy found that they have insulin resistance and glucose intolerance (Faulds et al., 2012; Kleber Costa Teixeira et al., 2018; Saengsirisuwan et al., 2009). Here, we show for the first time that glucose curve during GTT in 18‐month‐old female rats is similar to younger counterparts (Figure 2b,c). The absence of changes in glycemia in 12‐ and 18‐month‐old female rats may be related to the high concentration of PRL, as it has been reported that chronic moderate hyperprolactinemia is associated with increased release of insulin induced by a glucose load (Fleenor & Freemark, 2001; Reis et al., 1997). In addition, pancreatic islets from rats treated previously with dexamethasone had increased glucose‐stimulated insulin secretion (Fichna & Fichna, 2017; Rafacho et al., 2011). Thus, elevated plasma CORT and PRL concentrations in 18‐month‐old female rats (Figure 5d,e) could stimulate insulin secretion after a glucose load, countering the effect of estrogen deficiency, without change in glycemia values at this age. In addition, studies have found that PRL promotes systemic insulin sensitivity in obese rodents and humans (Ruiz‐Herrera et al., 2016).

Moreover, in the feeding animals, the highest glycemia in 3‐month‐old females could be a consequence of their low level of estrogen, since estradiol deficiency, as seen in aged females, is known to decrease insulin sensitivity in the liver, skeletal muscle, and white adipose tissue; reduce GLUT4 expression in skeletal muscle and white adipose tissue; and stimulate food intake and gluconeogenesis (Foryst‐Ludwig & Kintscher, 2010; Lu et al., 1998). The 18‐month‐old females in ad libitum conditions exhibited low plasma glucose, insulin, and triglyceride concentrations, indicating a possible decrease in intestinal absorption of glucose and triglycerides (Hahn et al., 1978; Hauge‐Evans et al., 2015; Krejs et al., 1980; Wahren & Feling, 1976).

The increased PRL concentration observed in older females may be due to the lack of response of hypothalamic tuberoinfundibular dopaminergic neurons or to a possible alteration of PRL receptors (Reymond, 1990). On the other hand, sexual differences found in plasma CORT levels are established by the neonatal estrogenization of females affecting different regulatory substrates of the HPA, including the gene expression of the CRH, arginine vasopressin, and hypothalamic glucocorticoid receptor (GR) (Patchev et al., 1995). However, the increased CORT in older females is due to the loss of negative feedback, caused by the decrease in GR in different parts of the brain related to HPA axis control and possibly to PRL stimulation, since it was shown that PRL stimulates CORT release in vitro (Lo et al., 1999, 2006; Lo & Wang, 2003; Mizoguchi et al., 2009).

Triglyceride plasma levels in 2‐month‐old females were lower than males (Figure 3a), possibly because females have a higher VLDL‐TG clearance rate, as already described in humans (Magkos et al., 2007; Palmisano et al., 2018; Wang et al., 2011). However, the sex difference in TG disappeared in the following months, which may be why females became obese and in this condition the VLDL‐TG clearance rate decreased, making TG values similar between females and males (Mittendorfer et al., 2003).

Sex differences found in 18‐month‐old feeding animals in serum TGs and free fatty acid concentrations may be because females have a higher clearance of free fatty acids in the muscle and therefore less formation of TGs in the liver (Palmisano et al., 2018). In addition, in the fed state, the lipoprotein lipase (LPL) synthesis is increased in the adipose tissue, and CORT could enhance LPL activity, promoting fatty acid uptake and storage in adipocytes (Ling et al., 2003; Oliver & Perenna, 1993; Peckett et al., 2011). Thus, the 18‐month‐old female rats exhibited higher plasma CORT than respectively male, and old female rats showed lower free fatty acid concentration than old male. In the case of total cholesterol, the sexual differences could be a consequence of a higher density of LDL receptor in the liver of females (Nanjee & Miller, 1988).

In reproductive age, it is known that women and females mice show higher HDL cholesterol level than men and male mice, respectively (Klingel et al., 2017; Link et al., 2015; Wang et al., 2011). These sexual differences could be related with the fact that females have two X chromosomes, which increased the expression of genes escaping X‐inactivation (Link et al., 2015).

Age differences observed in total cholesterol may be due to the fact that with aging LDL levels increase, because the circulating LDL clearance rate decreases by decreasing LDL hepatic receptor (LDLr) expression (Bose et al., 2005; Morgan et al., 2016). LDLr deficiency doubles total cholesterol increase (Morgan et al., 2016). Moreover, 3‐hydroxy‐3‐methylglutaryl coenzyme A reductase (HMGR), which is the limiting enzyme for cholesterol biosynthesis, increases its activation in aging (Pallottini et al., 2006).

Although increased leptin levels in adult animals are associated with increased fat, with aging, leptin gene expression increases independently of increased adiposity, so the development of hyperleptinemia is one of the characteristics observed during aging (Carrascosa et al., 2009; Kristensen et al., 1999; Li et al., 1997; Schautz et al., 2012). Moreover, aging leads to peripheral and central resistance to leptin (Carrascosa et al., 2009; Fernández‐Galaz et al., 2001; Scarpace & Tümer, 2001).

Plasma adiponectin concentrations are higher in adult females than in adult males, because testosterone selectively reduces adiponectin secretion of high molecular weight by adipocytes. The high adiponectin level in older male rats could be due to decreased androgens with age (Isobe et al., 2005; Nishizawa et al., 2002; Rosario et al., 2009; Smith et al., 1992; Xu et al., 2005).

Sex difference observed in plasma TSH concentration could be because the estrogens stimulate in the anterior pituitary activity of the type 1 deiodinase enzyme, whereas testosterone reduces it (Lisbôa et al., 2001). Estrogens also increase nuclear T3 density and TRH receptors in thyrotropic cells (Donda et al., 1990).

On the other hand, sex difference observed in plasma T3 concentration in adult animals was possibly due to the inhibitory effect of CORT on T3 production (Nunes et al., 2005). As discussed earlier and as observed in our results, plasma CORT concentration is higher in females than in males. Thus, in the absence of CORT inhibitory effect, T3 plasma concentration becomes higher.

In summary, our work demonstrated that the aging process induces an alteration in energy metabolism mainly in male rats and changes in plasma hormone concentrations that may or may not depend on sex. Although, previous results in the literature have described the effect of aging on energy metabolism in male rats, information about old females is scarce. Thus, our results show that the effects of aging on energy metabolism in rats are sex specific, with a better lipid profile and glucose tolerance in aged females.

## CONFLICT OF INTEREST

We have no conflict of interest to disclose.

## AUTHOR CONTRIBUTION

Susana Quirós Cognuck: Principal investigator who proposed the hypothesis, was involved in planning the experiments to test the hypothesis, participated in execution of all experiments, analyzed the data, created the figures, wrote the manuscript and is the corresponding author. Wagner Luiz Reis: He was involved in project planning, execution of some experiments, and reviewed the final version of manuscript. Marcia S Silva and Lucas Kniess Debarba: They participated in some experiments. André de Souza Mecawi: He was involved in project planning and reviewed the final version of manuscript. Francisco José Albuquerque de Paula: He was involved in determination of the body composition using DEXA. Celso Franci: He measured PRL. Lucila Leico Kagohara Elias: She participated in execution of some experiments and reviewed the final version of manuscript. José Antunes‐Rodrigues: He was involved in project planning, execution of some experiments, and reviewed the final version of manuscript.
